# Video and Wearable Sensor Technologies for Early Detection of Cerebral Palsy in Infants: A Scoping Review

**DOI:** 10.3390/jcm15041510

**Published:** 2026-02-14

**Authors:** Charlotte F. Wahle, Aura M. Elias, Nora A. Galoustian, Teana M. Tee, Michaela L. Juels, Christine Amacker, Heather Waters, Rachel M. Thompson

**Affiliations:** 1 David Geffen School of Medicine, University of California, Los Angeles, CA 90095, USA; cwahle@mednet.ucla.edu (C.F.W.); amelias@mednet.ucla.edu (A.M.E.); ngaloustian@mednet.ucla.edu (N.A.G.); 2 Herbert Wertheim College of Medicine, Miami, FL 33199, USA; teanatee24@gmail.com; 3 Department of Orthopaedic Surgery, University of California, San Francisco, CA 94143, USA; michaela.juels@ucsf.edu; 4 Department of Orthopaedic Surgery, Rady Children’s Hospital, San Diego, CA 92123, USA; camacker1@rchsd.org (C.A.); hwaters@rchsd.org (H.W.); 5 Department of Orthopaedic Surgery, University of California, San Diego, CA 92037, USA

**Keywords:** cerebral palsy, General Movements Assessment, pre-ambulatory, accelerometry, motion sensor, deep learning

## Abstract

It is well established that early diagnosis and subsequent intervention can result in significant benefits in infants with neurodevelopmental disorders such as cerebral palsy (CP). This scoping review aimed to assess the current state of the literature regarding the use of innovative and emerging technologies for early CP screening, diagnosis and phenotyping in pre-ambulatory children. Searches were performed across PubMed, Embase and Cochrane databases; articles were screened by four independent reviewers at the title/abstract and full-text levels. Forty-eight studies met the inclusion criteria. The most frequently used modalities included wearable sensors (e.g., accelerometers, inertial measurement units) and video-based motion analysis. These movement-tracking systems were used to screen for a variety of pediatric-onset neurodevelopmental disorders and have been useful in quantifying spontaneous infant movements, detecting the absence or abnormality of fidgety movement, or identifying atypical motor patterns. Although CP was our primary focus, several studies applied a similar pipeline to autism spectrum disorder (ASD) and spinal muscular atrophy (SMA), underscoring broader relevance for early neurodevelopmental screening, diagnosing and phenotyping. Overall, technology-assisted motor assessment demonstrated promising feasibility and diagnostic potential; however, most studies are limited by small sample sizes, short follow-up durations, and heterogeneous validation methods. Given the benefits of early intervention and the emerging capabilities of wearable and video-based analytics, larger multi-site and longitudinal datasets are needed to support early diagnosis, risk stratification, and functional phenotyping in CP.

## 1. Introduction

Cerebral palsy (CP), the most common childhood motor disability, is typically diagnosed between 12 and 24 months of age, with milder cases often recognized beyond this age range [1]. Delayed diagnosis during this critical period of neurodevelopment limits opportunities for early intervention, despite evidence that earlier treatment can improve long-term functional outcomes. Historically, clinicians have relied on delayed motor milestones and abnormal tone or posture for diagnosis, resulting in diagnostic delays with limited specificity, lacking early phenotyping and the ability to project expected outcomes during the pre-ambulatory period. Within early detection pathways, digital tools may support different clinical functions, including screening for atypical motor development, risk stratification for cerebral palsy, and assisting with diagnostic confirmation when paired with established clinical standards.

Early signs of CP, such as atypical general movements, are often subtle and require expert assessment. The Prechtl General Movements Assessment (GMA), which relies on visual perception of spontaneous movements—particularly “fidgety movements” between 9 and 20 weeks post-term—has become standard practice for early CP prediction with a sensitivity of 95–98% [2,3]. In combination with a structural brain MRI, the GMA is widely used for early detection of CP, highlighting the vital importance of movement analysis in this age group [1]. However, widespread implementation of the GMA remains constrained by the need for specialized training and certified raters, limited availability in many healthcare systems, and challenges with access for rural families [4]. These limitations highlight the need for scalable, objective tools that can support early risk stratification and triage.

Several emerging technologies aim to bridge these diagnostic gaps by enabling continuous and/or serial, objective quantifications of spontaneous infant movements. Wearable movement sensors, accelerometers, and computer vision-based video analysis have become increasingly feasible due to advances in hardware miniaturization, pose estimation, and machine learning, which now allow infant and pediatric motor patterns to be captured and analyzed using consumer-grade cameras and lightweight wearable devices. Several studies have demonstrated the feasibility and accuracy of these tools in identifying early signs of CP and other neurodevelopmental disorders. For instance, Airaksinen et al. introduced a smart jumpsuit with integrated sensors that achieved human-equivalent performance in classifying infant posture and movement patterns [4]. Similarly, Groos et al. validated a deep learning model capable of predicting CP from a single video clip of spontaneous movements with accuracy comparable to expert-administered GMA [5]. By quantifying spontaneous movements during the first months of life, these technologies may facilitate earlier screening and risk stratification before traditional diagnostic confirmation. However, despite promising early results, these approaches vary widely in sensor configurations, analytic methods, and validation standards, and few have undergone multi-site or longitudinal clinical validation.

Similar analytic methods and computational pipelines have been explored in other neurodevelopmental and neuromuscular conditions, including autism spectrum disorder (ASD), attention deficit hyperactivity disorder (ADHD), and spinal muscular atrophy (SMA). For example, Wilson et al. found that reduced movement variability—measured using wearable accelerometers—can predict ASD diagnoses as early as 18 months of age, underscoring the broader potential of such tools for neurodevelopmental surveillance and screening for variable developmental disorders at once [6]. Reich et al. further demonstrated the use of video-based skeletal tracking to detect age-specific motor patterns, achieving 88% accuracy in classifying fidgety versus non-fidgety movements using a shallow neural network [7]. SMA studies likewise have employed wearable or kinematic assessments to quantify early motor weakness. Although these conditions differ fundamentally from CP, their inclusion in the technological landscape highlights the broader methodological applicability of digital motor phenotyping and informs future multimodal screening approaches.

These emerging tools highlight the potential for objective and scalable motor phenotyping during the pre-ambulatory period, which could enable earlier screening and risk stratification in infants at elevated risk for cerebral palsy and related disorders. This could pave the way for personalized early interventions. The objective of this scoping review is to map the current landscape of digital video-based and wearable-sensor technologies used for early motor assessment in infants aged 0–36 months, with particular emphasis on clinical pathways relevant to early CP detection. Specifically, we aim to (1) characterize technological and analytic approaches, (2) evaluate diagnostic and feasibility characteristics relative to clinical reference standards, and (3) identify gaps, challenges, and future directions for clinical translation of digital motor phenotyping.

## 2. Methods

### 2.1. Search Strategy

A scoping review of the literature was performed in accordance with PRISMA 2020 guidelines for scoping reviews [8]. A formal protocol was not registered (e.g., PROSPERO) due to the exploratory nature of the topic. Searches were conducted across Pubmed (National Library of Medicine, MD, USA), Embase (Elsevier, Amsterdam, The Netherlands), and Cochrane (Wiley, Hoboken, NJ, USA) databases. The initial search was performed using the Boolean phrase: (“accelerometer” OR “wearable device” OR “sensor” OR “motion” OR “video”) AND (“pre-ambulatory” OR “non-ambulatory” OR “infants” OR “toddlers”) AND (“diagnosis” OR “pathology” OR “cerebral palsy” OR “neurological disorder” OR “motor disorder” OR “movement disorder” OR “neurodevelopmental disorder” OR “neurodevelopmental disability” or “neurodevelopment”) AND (“assessment” OR “evaluation” OR “detection”). Results were limited to studies from 2005 to 2024 to reflect the modern emergence of machine learning, pose estimation, and consumer sensing technologies.

### 2.2. Eligibility Criteria

Inclusion criteria encompassed original research involving pre-ambulatory patients (up to 36 months of age) that investigated the use of technology to assess movement, motor abilities, and/or neural development in the context of early diagnosis or risk stratification for cerebral palsy. Eligible “technology-based” studies included those utilizing sensing or automated computational analysis rather than subjective human observation alone. Technologies included (1) wearable sensors (e.g., accelerometers, gyroscopes, inertial measurement units, (2) instrumented laboratory-based motion analysis systems (e.g., multi-camera motion capture systems), (3) pressure or force sensors, and (4) non-instrumented video-based systems (e.g., smartphone or consumer RGB cameras) employing automated analytics pipelines. Eligible video-based studies were those employing automated analytics (e.g., pose estimation, kinematic feature extraction, skeletal tracking, machine learning classification, or other algorithm-based analysis techniques). Manual video review without automated computational analysis was excluded. Studies were included regardless of whether infants were typically developing, atypically developing, or of unknown developmental status.

Exclusion criteria included studies that lacked full text, were not published in English, focused solely on therapeutic interventions without diagnostic or screening components, involved only clinical observational scoring without technological augmentation, and focused exclusively on non-CP neuromuscular diseases without methodological relevance. The restriction to English-language publications was based on feasibility constraints for screening and data extraction. While cerebral palsy was the primary clinical target, studies involving ASD, ADHD, SMA, or non-specific high-risk cohorts were included only when early motor behavior was analyzed using comparable video- or sensor-based methodologies and within the same pre-ambulatory age range. These studies were included to describe methodological approaches to early motor assessment, rather than as evidence for diagnostic equivalence with CP.

### 2.3. Screening and Data Extraction

Title/abstract and full-text screening were performed in two phases in Covidence (Veritas Health Innovation, Melbourne, Australia; Available at www.covidence.org.) by four independent reviewers (C.W., N.G., M.J., A.E.). Each article was screened by at least two reviewers, with disagreements resolved by a fellowship-trained pediatric neuromuscular specialist (R.T.).

**Figure 1:**
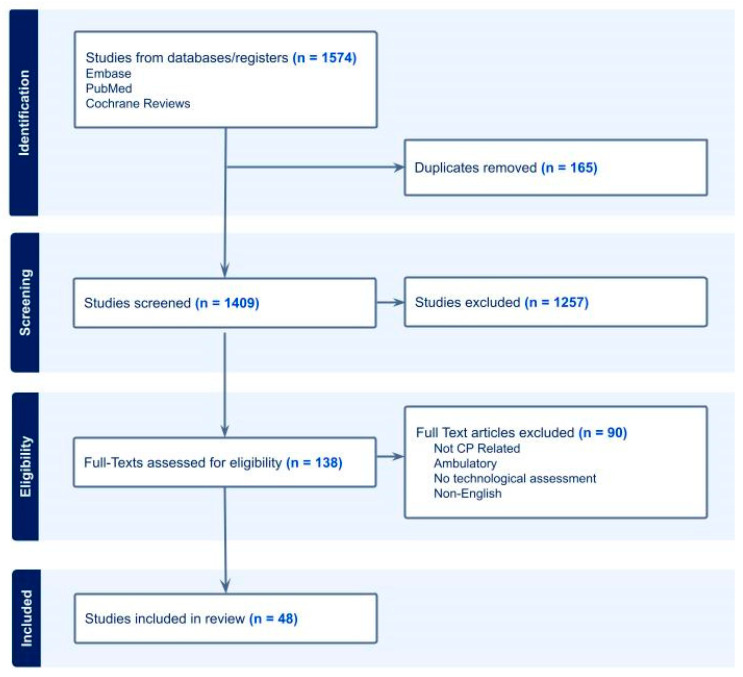
PRISMA flow diagram outlining the study identification, screening, eligibility, and inclusion process.

For studies meeting final eligibility, descriptive and methodological data were extracted using a structured template. Extracted variables included study design and setting, participant characteristics and age at assessment, pathology or clinical target, technology modality, analytic approach, clinical reference standard, validation setting, performance metrics, and feasibility and scaling notes. Discrepancies in extracted data were resolved through consensus among the extraction team with adjudication by R.T. when needed. Following extraction, studies were classified for synthesis into two domains: CP-relevant studies (studies evaluating CP diagnosis, CP risk, or fidgety movement classification) and non-CP motor phenotyping studies (ASD, SMA, high-risk infants), included for methodological context. A total of forty-eight studies met the inclusion criteria and were included in the scoping review. The study identification, screening, eligibility assessment, and inclusion process are summarized in a PRISMA-style flow diagram in Figure 1.

### 2.4. Validation and Risk of Bias Appraisal Approach

To characterize the methodological rigor and translational maturity of CP-relevant digital motor assessment studies, we performed a structured descriptive appraisal focusing on validation-related domains relevant to digital diagnostic technologies. Traditional diagnostic risk-of-bias tools such as QUADAS-2 or RoB-2 are designed for studies evaluating diagnostic accuracy with a clearly defined index test and reference standard [9]. Several included studies in this review investigated feasibility, feature extraction, or early risk stratification without a formal diagnostic endpoint, rendering such domains non-applicable.

For this reason, we adopted a QUADAS-aligned descriptive appraisal focusing on dimensions relevant to digital motor phenotyping (validation setting, presence of external validation, sample size adequacy, cohort representativeness, clinical reference standard quality, follow-up duration, and binding/reporting transparency). This approach was chosen to reflect the heterogeneity and early developmental stage of the literature and enables the comparison of methodological rigor and translational readiness without inappropriately forcing studies into a diagnostic accuracy framework. This appraisal was not intended to generate a summary risk-of-bias score but rather characterize validation maturity and translational readiness across studies.

## 3. Results

### 3.1. Study Characteristics and Modalities

A total of 48 studies met the inclusion criteria, comprising 33 video-based [5,7,10,11,12,13,14,15,16,17,18,19,20,21,22,23,24,25,26,27,28,29,30,31,32,33,34,35,36,37,38,39,40] and 15 wearable sensor-based [4,41,42,43,44,45,46,47,48,49,50,51,52,53,54] approaches for early motor assessment in pre-ambulatory infants. Following full-text data extraction, we identified a subset of twenty-three studies that directly investigated cerebral palsy (CP) or fidgety movement (FM) classification, of which nineteen were video-based, and four were wearable sensor-based. The characteristics of the CP-specific studies (*n* = 23) are summarized in Table 1.

Most studies assessed infants within the fidgety movement period (9–20 weeks post-term), although age ranges varied across modalities and study objectives. For synthesis, findings are organized across four domains aligned with the review question: (1) diagnostic performance, (2) technological validation, (3) feasibility and scalability, and (4) clinical role.

### 3.2. Diagnostic Performance Relative to Clinical Standards

Twenty-seven studies assessed diagnostic performance with respect to clinical reference standards. Among video-based studies, fifteen evaluated agreement with expert-assigned classifications, with a majority investigating GMA classifications or absence/presence of fidgety movements (FM) [7,11,13,16,17,26,27,28,29,30,33,36] and the remainder compared with other clinical assessments [31,50,52]. Such studies directly compared algorithm-derived categorizations with clinician ratings using metrics such as Cohen’s κ, overall accuracy, percent agreement, and sensitivity. Sensitivity ranged from 82 to 90%, and classification accuracy ranged from 83 to 90%. Reported agreement was generally moderate to high, although specific values varied depending on study design and population.

Sixteen studies [5,10,11,12,15,17,20,22,23,27,29,35,37,43,44,54], primarily those that utilized data collected via video capture, evaluated predictive validity against a formal diagnosis of CP or neurodevelopmental disorder diagnosis. Reported sensitivities were generally high, ranging from 81 to 93%, with one study reporting a considerably lower sensitivity of 55% [35]. Specificity ranged from 71 to96%. Follow-up time to a confirmed CP diagnosis in such studies were generally between 1 and 2 years. Reported performance metrics relative to clinical reference standards, including sensitivity, specificity, accuracy, and validation endpoints for CP-relevant studies, are presented in Table 2 to facilitate cross-study comparison.

Studies employing wearable sensors less frequently employed clinical outcomes as endpoints; most reported feasibility outcomes, motor quantification metrics, or group-level differences without predictive modeling. Only a minority attempted FM classification or CP risk stratification, and none incorporated multisite validation or long-term follow-up.

### 3.3. Technological Validation and Interpretability

Across all 45 included studies, data acquisition and analytical pipelines exhibited substantial methodological heterogeneity. Video-based studies used standard RGB cameras, smartphone cameras, RGB-D sensors, or multi-camera motion capture systems. Analytical pipelines commonly involve pose estimation or skeletal tracking, kinematic or spatiotemporal feature extraction, and machine-learning or deep-learning classification.

Studies used accelerometers, IMUs, gyroscopes, or EMG/force sensors, with sensors mounted on the lower or upper extremities, trunk, or via full-body suits to quantify limb coordination, muscle activation, and spatiotemporal motor patterns. Sensor configurations ranged from single-site placements to multi-sensor systems. Feature extraction varied considerably; some relied on predefined kinematic and spatiotemporal features derived from movement trajectories, whereas others used deep learning models to automatically learn features directly from raw video or sensor data.

Across modalities, analytical methods included classical machine-learning models (e.g., SVMs, random forests) as well as deep neural networks. Internal validation was predominant, often using cross-validation or train/test splits.

### 3.4. Feasibility and Scalability in Real-World Settings

While no studies directly measured feasibility or scalability outcomes, related information was reported descriptively within Section 4 of each study. Ease of application in both the home and clinic was commonly reported amongst those who investigated video-based data [27,29,38]. Passmore et al. utilized smartphone cameras, in which participants collected data at their respective homes [26]. Other studies using dedicated cameras described small and easily assembled clinic setups [7,28,46]. Challenges included variable lighting, occlusion, quality, framing, and camera distance, as well as variation in clothing and environment, which affected data quality and downstream processing [19,29,33,40]. Remote video capture occasionally raised privacy and data-handling considerations [26,55].

Studies investigating wearable sensors were predominantly collecting data outside of the hospital setting to capture more naturalistic movements [45,49,54]. Investigators reported that monitors distracted some infants, and the reproducibility of sensor placement and calibration was noted as a challenge [4,50,54]. Additional issues included device hygiene, battery management, and occasional hardware malfunction [51,53].

Across both video and wearable modalities, authors frequently commented on the feasibility of application in clinical environments due to the relative affordability and accessibility of the underlying hardware, although formal metrics were not reported in any study.

### 3.5. Clinical Role of Sensor- and Video-Based Technologies

Across all included CP-relevant studies, automated movement analysis methods were intended primarily for early identification and risk stratification rather than definitive diagnosis. Early identification/risk stratification was operationalized through: (1) automated fidgety movement (FM) classification, (2) detection of abnormal movements, and (3) stratification of infants with known perinatal risk factors. Several studies described their proposed approaches as intended for initial screening only or as complements to existing clinical screening assessments. Støen et al. described a triage-based application of automated movement analysis, in which algorithm-derived outputs were used to determine whether observational GMA should be performed. Under this approach, a subset of infants was identified for referral to early intervention without additional observational GMA [17]. Generally, the aim in all included studies was to augment current early detection pathways using scalable and accessible technology rather than to replace current algorithms and clinical evaluations. To contextualize the operational and analytic differences between video-based and sensor-based systems, a modality comparison summary is provided in Table 3.

### 3.6. Risk of Bias and Validation Appraisal

Validation characteristics across CP-relevant studies are summarized in Table 4, including validation setting, external validation status, sample size category, reference standard quality, cohort representativeness, follow-up duration, and reporting of blinding or reviewer independence.

Most studies employed prospective designs with internal validation only. External validation using independent datasets was rare and observed primarily in randomized or multi-site studies. Sample sizes varied widely, with many studies categorized as small or pilot investigations. Clinical reference standards most commonly included expert GMA or later CP diagnosis, although reference-standard quality and reporting varied across studies. Follow-up duration ranges from no longitudinal follow-up to several years, with longer follow-up more frequently observed in studies evaluating CP prediction rather than early motor phenotype classification. Reporting of blinding or reviewer independence was inconsistent.

### 3.7. Applications Beyond Cerebral Palsy

Although many included technologies were designed to support CP screening or fidgety movement (FM) classification, several studies applied similar computational pipelines to other specific neurodevelopmental contexts. These included both general neurodevelopmental disorder (NDD) cohorts without a named endpoint and specific diagnostic groups such as autism spectrum disorder (ASD) and spinal muscular atrophy (SMA). These studies were retained to contextualize methodological generalizability across early neurodevelopmental phenotypes but were not included in CP-specific diagnostic synthesis or risk-of-bias appraisal.

#### 3.7.1. General NDD and Developmental Motor Profiling Studies

Twenty studies [7,16,19,24,25,28,30,33,36,37,39,40,41,42,45,46,48,49,50,51] examined movement patterns in general neurodevelopmental delay cohorts without CP or fidgety movement endpoints. These included preterm infants, high-risk neonatal follow-up cohorts, infants with heterogeneous developmental delays, and normative developmental surveillance samples. Outcomes commonly focused on typical vs. atypical motor pattern differentiation, developmental trajectory mapping, limb coordination or variability indices, and spatial–temporal kinematic profiling. Although these studies did not address CP diagnosis or FM classification, they employed similar analytical pipelines, indicating methodological portability across neurodevelopmental populations.

#### 3.7.2. Specific Diagnoses Studies (ASD, SMA)

Five studies targeted specific neurodevelopmental conditions. Three ASD studies demonstrated that abnormalities in spontaneous movement, motion complexity, or kinematic variability may precede behavioral symptom emergence. For example, Baccinelli developed a markerless video tool (Movidea) for early ASD risk assessment using network-based video dataset [24]. Doi et al. analyzed spontaneous bodily movements in infants at 4 months of age with clinical ASD follow-up at 18 months of age [32]. Wilson et al. reported lower accelerometry-derived “motion complexity” in infants later diagnosed with ASD [47]. Two SMA studies evaluated early neuromuscular signatures, finding correlations between digital motor metrics and standardized clinical motor assessments [47,53].

## 4. Discussion

This scoping review provides a comprehensive synthesis of recent literature on contemporary video-based and wearable-sensor approaches for early neuromotor assessment in pre-ambulatory infants, with particular focus on cerebral palsy. While the General Movements Assessment (GMA) remains a highly sensitive and well-validated predictor of CP, the specialized training required and limited global availability create structural barriers for widespread early screening. Emerging technologies such as smartphone video capture, accelerometry, and multi-sensor motion tracking demonstrate promising capacity to support early identification, timely intervention, and more efficient referral pathways [1,46]. These modalities, therefore, represent a potential avenue for improving equitable access to early detection and intervention services. Although predictive performance from included studies is encouraging, the broader scientific and clinical landscape reveals meaningful gaps in validation, scalability, and equitable access that must be addressed before these tools can be integrated into routine care.

### 4.1. Diagnostic Precision and Validation Gaps

Across CP-relevant studies, automated video-based assessments demonstrated encouraging diagnostic performance relative to expert GMA ratings, and several achieved promising sensitivity and specificity for predicting later CP diagnosis at 12–24 months of age. However, strong diagnostic performance alone is insufficient for clinical deployment. Two critical validation gaps emerged: (1) limited longitudinal follow-up and (2) lack of external validation across independent settings. These limitations constrain generalizability and contribute to a well-described pattern in digital health tools in which high performance within development cohorts does not translate across health systems, populations, or environments.

Wearable motion sensors contribute valuable biomechanical information, but their translation into clinical practice remains limited by workflow requirements, calibration demands, infant compliance, and occasional hardware malfunction [4,14,39]. Concerns have also been raised regarding whether external sensors alter natural motor behavior. Moreover, variable compliance and hardware malfunction may introduce measurement artifacts. By comparison, video-based methods negate most hardware/sensor-related concerns and align more readily with existing CP screening pathways, particularly given the rise in remote and telemedicine-based infant assessments. This method offers the opportunity to help reduce barriers to accessing care, particularly in low-resource areas where certified GMA assessors may be unavailable [37,40,46]. However, variability in data collection methods and quality introduces analytic challenges not typically encountered in controlled research settings.

Another meaningful concern relates to how risk is conceptualized. Many studies framed early detection as a binary classification problem (e.g., presence vs. absence of FM) rather than modeling neurodevelopment as a trajectory. Binary framing fails to capture the clinical heterogeneity of early neurodevelopmental disorders, limits nuanced risk stratification, and restricts the ability to support personalized intervention planning.

Finally, sample sizes across nearly all CP-focused studies were modest, limiting the statistical power of their findings and the generalizability of reported results. When combined with short follow-up windows, this prevents meaningful assessments of long-term prognostic value. While many technologies demonstrated strong screening performance in infancy, few tracked motor or functional outcomes beyond the early years of life, making it difficult to determine whether early digital phenotypes predict later disability or treatment response.

### 4.2. Clinical Role: Screening and Triage Rather than Diagnostic Confirmation

Although much of the literature reports diagnostic performance, the realistic clinical value of these technologies lies within their screening, training, and referral optimization capabilities. In their current state, most digital motor assessment tools lack the external validation, standard workflows, and longitudinal outcome tracking required for standalone diagnostic use.

Instead, these technologies are best conceptualized as adjunctive screening tools that augment existing clinical pathways rather than as replacements for expert assessment. When deployed upstream, digital tools may help identify infants who warrant expedited referral for formal evaluation, including expert GMA, neuroimaging, or subspecialty consultation. This role is particularly relevant in settings where access to certified GMA assessors is limited or could be delayed.

Additionally, digital screening tools may support clinical training and decision support by providing objective, reproducible movement features that complement clinical judgment. Framing these technologies as tools for early risk stratification, rather than as definitive diagnostics, aligns their current capabilities with relevant clinical needs and mitigates the risk of inappropriate use of premature technology and algorithms.

### 4.3. Technological Modalities, Feasibility, and Scalability Considerations

The feasibility and scalability of digital motor assessment tools go beyond their performance and depend on their ability to fit within the real-world clinical and community workflow. Video-based methods may provide the greatest feasibility to translate into practice within the clinic and community. Clinicians and caregivers are often equipped with smartphones that have video capture capabilities and can deploy the use of this technology to capture videos of infants for early risk identification. This would allow infants to be assessed for risk spanning a variety of settings from acute inpatient to the outpatient clinic and the community, including rural or low-resource areas. Research is needed to determine best practices in regard to clothing, background, lighting, subject position, camera positioning and interaction with the environment. Further, in order to gather adequate video footage, the participating clinician or caregiver must have an understanding of the criteria for capturing a high-quality, interpretable video. Privacy and data-handling considerations are also at play. Video capture must meet healthcare settings’ high standards for the protection of patient health information, which must also be taken into consideration.

Moreover, while wearable sensors may provide informative biomechanical data, there is less ease of use in a real-world setting. This may be a particularly inaccessible technology in more low-resource areas. On top of these scalability restrictions, there are barriers pertaining to calibration requirements, hardware malfunction and reproducibility of sensor placement protocols. There are also concerns of restrictions secondary to donning an external device on the infant, impacting their natural movements within their environment. These varying factors lend themselves to less feasibility and scalability in a real-world setting.

If these barriers can be addressed, both video and wearable-sensor-based technologies show promise toward enabling early screening of infants at risk for cerebral palsy on a grand scale. Earlier, inclusive screening and risk stratification will allow for targeted clinical follow-up and potentially early intervention with the goal of improved function. While more work must be carried out to validate results in larger clinical settings and field-test these technologies, utilization of one or more of these emerging technologies as a screening tool is feasible in the short term, with their potential use as a diagnostic tool as limitations continue to be addressed in the long term.

### 4.4. Implications of Artificial Intelligence, Machine-Learning Algorithms, Dataset Bias, and Equity Implications

Many of the included studies rely on artificial intelligence (AI) and machine learning analytical tools for data analysis [5,7,15,22,29,33,39,40]. AI and machine-learning algorithms have emerged as valuable tools in the early detection and diagnosis of neurodevelopmental disorders such as cerebral palsy [5,13,15,22]. The increasing availability of free or low-cost artificial intelligence tools presents significant opportunities to enhance diagnostic analysis and accessibility. These technologies leverage insights from prior patient data and reduce barriers to accessing timely diagnosis [5,7].

However, the effectiveness and equity impact of AI is heavily dependent on the quality and diversity of the data on which it is trained. In many cases, the included study populations were limited to either high-risk or low-risk infants, with few including typically developing control subjects [10,11,14,25,26,45]. In addition, the tools and algorithms were typically trained and tested within single-site datasets and rarely evaluated in independent or demographically diverse cohorts, limiting the assessment of generalizability across healthcare systems, geographic regions, and socio-economic contexts.

These limitations raise important concerns regarding algorithmic bias and equity. AI models trained on homogenous populations may perform poorly when applied to infants from underrepresented racial, socioeconomic or geographic groups, potentially exacerbating existing disparities in early CP detection rather than mitigating them. Without deliberate inclusion of diverse cohorts and external validation across varied clinical settings, there is a risk that AI-driven screening tools will preferentially benefit populations already well served by subspecialty care [55].

To ensure equitable translation, future development of AI-based motor assessment tools must prioritize dataset diversity, transparent reporting of cohort characteristics, and rigorous external validation. Only through intentional design and validation can AI-driven phenotyping fulfill its potential to support earlier, more equitable identification of infants at risk for cerebral palsy and other related neurodevelopmental conditions.

### 4.5. Implications of Digital Motor Assessment Beyond Cerebral Palsy

A notable proportion of included studies examined digital motor assessment in broader neurodevelopmental or neuromuscular conditions beyond cerebral palsy. While these studies were not designed to evaluate CP diagnosis or fidgety movement classification, they provide important insight into the methodological generalizability and translational potential of digital motor phenotyping.

Across non-CP studies, investigators employed analytic pipelines highly comparable to those used in CP-focused research, including automated video-based pose estimation, kinematic feature extraction, accelerometer-derived variability metrics, and machine learning-based classification. Collectively, these studies suggest that digitally captured early movement features reflect global neurodevelopmental integrity, rather than condition-specific pathology, with diagnostic specificity emerging later in development. Across developmental contexts, deviations in movement complexity, coordination, or variability consistently preceded overt clinical or behavioral symptom onset, reinforcing the premise that early motor behavior serves as an integrated marker of neurological function during critical developmental windows.

These observations reinforce the premise underlying CP-focused work: that quantitative movement analysis may enable earlier identification of atypical neurodevelopment before traditional clinical diagnostic thresholds are met. At the same time, they underscore the importance of distinguishing screening utility from diagnostic confirmation. While the portability of analytic frameworks across conditions is promising, condition-specific validation, outcome alignment, and longitudinal follow-up remain essential before clinical adoption.

Taken together, the literature supports a future in which digital motor phenotyping serves as an early, cross-condition screening layer, with downstream diagnostic clarification guided by disorder-specific clinical standards. Realizing this vision will require careful separation of screening utility from diagnostic claims, along with rigorous validation within each clinical context.

## 5. Future Directions

To bridge the gap between promising diagnostic performance and clinical adoption, future work should emphasize phenotyping frameworks capable of capturing developmental nuance beyond binary risk labels. Specifically, they should aim to develop more granular, categorical assessment models that reflect the spectrum of motor dysfunction. Integrating movement data with other biomarkers, such as genetic profiles or neuroimaging findings, may enhance diagnostic precision and open new avenues for personalized treatment. However, such advances require shared datasets, common acquisition standards, and coordinated validation efforts across institutions. In fact, Spittle et al. recently described an interdisciplinary consortium of clinicians from more than ten different countries across four continents who together built a state-of-the-art CP screening system with data and expertise from neurologists, pediatricians, neonatologists, researchers, engineers, and more, demonstrating the promise and value of broad, multi-site collaborations [3]. Finally, prospective, longitudinal research is needed to determine whether early alterations in motor signatures predict later developmental outcomes and whether early identification changes the timing or efficacy of interventions.

## 6. Limitations

This scoping review has several limitations. First, many included studies enrolled small cohorts, limiting statistical power and reducing confidence in performance estimates. Second, most studies were cross-sectional, lacking long-term trajectories to adequately reflect developmental variability over time. Third, binary outcome frameworks (e.g., presence vs. absence of CP or FM) were common, constraining opportunities for early phenotyping, severity grading, or a developmental trajectory model.

Methodologically, we restricted the review to English-language publications, which may introduce language and regional bias and reduce applicability to non-English speaking settings. This limitation was due to feasibility constraints in screening and data extraction. The inclusion of ASD, SMA, and other neurodevelopmental cohorts introduces diagnostic heterogeneity; however, these studies were retained for methodological context and were not interpreted as evidence of diagnostic equivalence with CP.

Despite these limitations, this review provides the first structured synthesis of contemporary video-based and wearable-sensor technologies for pre-ambulatory neuromotor assessment, offering a foundation for future translational and regulatory work.

## 7. Conclusions

Movement reflects the amalgamation of overlapping neurological, sensory, and muscular processes. Digitally quantifying these dynamics, whether via wearable or video-based technologies, offers a promising tool for earlier identification of deviations in neuromotor development. While this scoping review highlights various technologies available to assess neurotypical and atypical movement patterns, there is currently no single diagnostic test capable of identifying the breadth of neural and neurodevelopmental abnormalities or variations observed in early infancy.

As data infrastructure, computational tools, and telemedicine platforms continue to mature and become more ubiquitous, there is room to develop scalable technologies capable of supporting early detection and intervention for infants at risk of CP and other neurodevelopmental conditions. Realizing this goal will require collaborative, multidisciplinary efforts that integrate clinical expertise, engineering innovation, analytical depth, and strong implementation science to translate potential promise into meaningful improvements in early childhood neurodevelopmental care.

## Figures and Tables

**Table 1:** Characteristics of included studies relevant to cerebral palsy (*n* = 23). Studies are summarized by country, design, population characteristics, sample size, age at assessment, digital modality, clinical reference standard, evaluated motor outcome, and follow-up duration.

Author/Year	Country	Study Design	Population	*N*	Age	Modality	Reference Standard	Clinical Task	Follow-Up
Adde 2009 [11]	Norway	Prospective	Full-term + Preterm	82	10–18 wks	Video	GMA (expert)	FM Classification	No follow-up
Adde 2010 [12]	Norway	Prospective	Preterm	30	10–15 wks	Video	CP Diagnosis	CP Risk Stratification	5 yrs
Adde 2013 [13]	Norway	Prospective	Full-term + Preterm	52	9–17 wks	Video	CP Diagnosis	FM Classification + CP Prediction	2 yrs
Berger 2019 [21]	United States	Prospective	Full-term + Preterm	31	6–8.5 mos	Video	Motor Assessment	CP vs. TD Posture	No follow-up
Adde 2018 [18]	Norway	Prospective	Preterm	27	3–15 wks	Video	GMA (expert)	FM Classification	No follow-up
Groos 2022 [5]	Norway	RCT	Not Reported	557	9–18 wks	Video	CP Diagnosis	CP Prediction	1 year
Ihlen 2019 [22]	Norway	Prospective	Preterm	377	9–15 wks	Video	CP Diagnosis	CP Prediction	3.7 yrs
Karch 2012 [43]	Germany	Prospective	Not Reported	75	3 mos	Wearable	CP Diagnosis	Neurodeficit Prediction	2 yrs
Meinecke 2006 [10]	Germany	Prospective	Full-term + Preterm	22	Not Reported	Video	CP Diagnosis	CP Risk Stratification	2 yrs
Nguyen-Thai 2021 [29]	Australia	Retrospective	Not Reported	235	14–15 wks	Video	GMA (expert)	FM Classification	No follow-up
Orlandi 2018 [20]	Canada	Retrospective	Preterm	127	3–5 mos	Video	CP Diagnosis	CP Prediction	No follow-up
Passmore 2024 [38]	Australia	Prospective	Full-term + Preterm	341	Not Reported	Video	GMA (expert)	FM Classification	2 yrs
Philippi 2014 [14]	Germany	Prospective	Full-term + Preterm	67	2.5–3.5 mos *	Video	CP Diagnosis	CP & NDI prediction	2 yrs
Passmore 2020 [26]	Australia	Retrospective	Full-term + Preterm	510	Not Reported	Video	GMA (expert)	FM Classification	No follow-up
Prosser 2022 [34]	United States	Prospective	Full-term + Preterm	15	4–6.5 mos	Video	Motor Outcome at FU	Motor Impairment	2 yrs
Raghuram 2019 [23]	Canada	Retrospective	Preterm	152	IQR 24.4–27.7 wks	Video	Motor Outcome at FU	Motor Impairment	No follow-up
Raghuram 2022 [35]	Canada	Prospective	Preterm	252	26–29 wks	Video	CP Diagnosis	CP Prediction	2 yrs
Rahmati 2014 [15]	Norway	Retrospective	Not Reported	78	Not Reported	Video	CP Diagnosis	CP Prediction	2 to 5 yrs
Rahmati 2016 [44]	Norway	Prospective	Not Reported	78	10–18 wks	IMU	CP Diagnosis	CP Prediction	Up to 5 yrs
Schroeder 2020 [27]	Germany	Prospective	Preterm	29	14.8 ± 0.7 wks *	Video	GMA (expert)	FM Classification	1–2.6 years
Støen 2017 [17]	Norway	Prospective	Preterm	150	24–32.1 wks	Video	CP Diagnosis	CP Prediction	No follow-up
Verhage 2024 [54]	Netherlands	Prospective	Full-term + Preterm	50	3–12 mos	Wearable	Early Motor Assessment	Motor Asymmetry Detection	No follow-up
Von Gunten 2023 [52]	Switzerland	Prospective	Full-term + Preterm	8	9–12 mos	Wearable	Early Motor Assessment	CP Risk Stratification	6 weeks

Cerebral Palsy (CP); General Movements Assessment (GMA); Fidgety Movements (FM); Inertial Measurement Units (IMU); * Corrected age.

**Table 2:** Diagnostic performance of digital motor assessment studies relevant to cerebral palsy. Reported sensitivity, specificity, accuracy, outcome targets, analytic approaches, and follow-up durations are summarized across the included studies.

Author/Year	Reference Standard	Outcome Target	Outcome Type	Sensitivity	Specificity	Other Metrics	Follow-Up
Groos 2022 [5]	CP Diagnosis	CP Prediction	Binary	71.4%	94.1%	Accuracy 90.6%	1 year
Meinecke 2006 [10]	CP Diagnosis	CP Risk Stratification	Probabilistic risk score	-	-	Accuracy 73%	2 years
Adde 2009 [11]	GMA (Expert)	FM Classification	Ordinal (FM grade)	81.5%	70%	-	No follow-up
Adde 2010 [12]	CP Diagnosis	CP Risk Stratification	Probabilistic → Binary	85%	71%	-	Up to 5 years
Rahmati 2014 [15]	CP Diagnosis	CP Prediction	Binary	86%	92%	Accuracy 91%	Up to 5 years
Støen 2017 [17]	CP Diagnosis	CP Prediction	Binary/Probabilistic	90%	80%	-	No follow-up
Orlandi 2018 [20]	CP Diagnosis	CP Prediction	Risk Prediction	-	-	Accuracy 92%	No follow-up
Ihlen 2019 [22]	CP Diagnosis	CP Prediction	Binary	92.7%	81.6%	-	3.7 years
Raghuram 2019 [23]	Motor Outcome	Motor Impairment	Binary/ Multi-class	79% *	63%	Accuracy 66%	No follow-up
Schroeder 2020 [27]	GMA (Expert)	FM Classification	Agreement Metrics	-	-	κ = 0.78, ICC = 0.926	12 to 31 months
Nguyen-Thai 2021 [29]	GMA (Expert)	FM Classification	Ordinal (FM grade)	-	-	AUC 81.87%	No follow-up
Raghuram 2022 [35]	CP Diagnosis	CP Prediction	Binary	55% *	80%	-	2 years
Passmore 2024 [38]	GMA (Expert)	FM Classification	Ordinal (FM grade)	76 ± 15% *	-	-	2 years
Karch 2012 [43]	CP Diagnosis	Neurodeficit Prediction	Binary	90%	96%	-	2 years
Rahmati 2016 [44]	CP Diagnosis	CP Prediction	Binary	85%	92%	Accuracy 91%	Up to 5 years
Verhage 2024 [54]	Early Motor Assessment	Motor Asymmetry Detection	Continuous Motor Metric	-	-	AUC 0.88 –0.96	No follow-up

* Fidgety Movement (FM); General Movements Assessment (GMA).

**Table 3:** Comparative characteristics of video-based and wearable sensor technologies for early neuromotor assessment. Key domains relevant to clinical translation—including data sources, workflow requirements, interpretability, scalability, and validation focus—are contrasted to highlight modality-specific strengths and limitations.

	Video-Based	Wearable Sensor
Primary Data Source	2D/3D visual motion (RGB, RGB-D, skeletal pose)	Accelerometry, gyroscope, EMG, IMU angular velocity
Common Use Cases in CP	FM classification, GM quantification, CP risk prediction	Motor feature quantification, coordination metrics, and spatiotemporal analysis
Hardware Burden	Low (consumer camera or smartphone)	Moderate–High (multi-sensor systems, calibration equipment)
Environmental Sensitivity (Data Capture)	Sensitive to lighting, occlusion, framing, and distractions	Sensitive to motion artifacts, placement and attachment
Privacy & Data Requirements	Requires PHI-secure video storage; HIPAA compliance	Less identifiable data; fewer PHI concerns; device management protocols
Workflow & Training Requirements	Minimal for data capture (parent/clinician recorded)	Requires trained personnel for placement, calibration, and syncing
Infant Interaction & Tolerance	Passive, no hardware; preserves spontaneous movement	Device attachment may alter natural movements; potential discomfort or distraction
Scalability Potential	High; compatible with telemedicine and home capture	Moderate; limited by device cost, calibration, hygiene, and replacement
Interpretability	Whole-body kinematics when pose estimation is used	Fine-grained motion metrics; less intuitive without biomechanical models
Algorithmic Pathways	Pose estimation/optical flow → kinematic feature extraction → ML/DL classifier	Raw IMU/accelerometry signals → feature engineering → ML/DL sequence models
Validation Focus	More CP outcome-based validation and FM → CP prediction studies	Primarily, feasibility and motor quantification; Fewer CP outcome-based studies
External Validation	Limited but emerging multi-site datasets	Rare external validation; heavy reliance on internal cross-validation
Cost Considerations	Low hardware cost; software/compute main expense	Moderate-High hardware cost; recurring device/maintenance needs
Equity & Accessibility Considerations	Potential to reduce disparities via smartphone access & telehealth	May increase disparities without subsidy; device access varies
Best Contexts of Use	Remote screening, triage, FM detection, longitudinal follow-up	Controlled clinic/lab settings, detailed biomechanics, specific motor domain quantification
Key Practical Constraints	Video quality variance, occlusion, caregiver training, privacy regulations	Calibration, hardware malfunction, placement reproducibility, hygiene
Current Clinical Role	Augments early screening and risk stratification; complements observational GMA	Objective quantification of motor domains; complementary biomechanical assessment
Translational Readiness	Advancing toward clinical piloting in remote/telehealth contexts; requires regulatory evaluation	Early-stage research; limited clinical deployment; primarily prototyping and feasibility testing

**Table 4:** Methodological quality and validation characteristics of included studies relevant to cerebral palsy. Studies are appraised by validation setting, external validation, sample size, reference standard quality, cohort representativeness, follow-up duration, and reporting transparency.

Author/Year	Validation Setting	External Validation	Sample Size	Reference Standard Quality	Cohort Representativeness	Follow-Up Duration	Blinding/Reviewer Independence
Adde 2009 [11]	Prospective; Internal	No	Small	Moderate	Mixed	None	Not Reported
Adde 2010 [12]	Prospective; Internal	No	Pilot	High	High-Risk Only	Long	Not Reported
Adde 2013 [13]	Prospective; Internal	No	Small	High	Mixed	Standard	Not Reported
Berger 2019 [21]	Prospective; Internal	No	Small	Moderate	Mixed	None	Not Reported
Adde 2018 [18]	Prospective; Internal	No	Pilot	Moderate	High-Risk Only	None	Not Reported
Groos 2022 [5]	RCT	Yes (multi-site)	Adequate	High	Mixed	Short	Reported
Ihlen 2019 [22]	Prospective; Internal	No	Adequate	High	High-Risk Only	Standard	Reported
Karch 2012 [43]	Prospective; Internal	No	Small	High	Unknown	Standard	Not Reported
Meinecke 2006 [10]	Prospective; Internal	No	Pilot	High	Mixed	Standard	Not Reported
Nguyen-Thai 2021 [29]	Retrospective; Internal	No	Adequate	Moderate	Unknown	None	Reported
Orlandi 2018 [20]	Retrospective; Internal	No	Limited	High	High-Risk Only	None	Reported
Passmore 2024 [38]	Prospective; Internal	No	Adequate	Moderate	Mixed	Standard	Reported
Philippi 2014 [14]	Prospective; Internal	No	Small	High	Mixed	Standard	Not Reported
Passmore 2020 [26]	Retrospective; Internal	No	Adequate	Moderate	Mixed	None	Reported
Prosser 2022 [34]	Prospective; Internal	No	Pilot	Moderate	Mixed	Standard	Reported
Raghuram 2019 [23]	Prospective; Internal	No	Limited	Moderate	High-Risk Only	None	Reported
Raghuram 2022 [35]	Prospective; Internal	No	Adequate	High	High-Risk Only	Standard	Reported
Rahmati 2014 [15]	Prospective; Internal	No	Small	High	Unknown	Long	Reported
Rahmati 2016 [44]	Prospective; Internal	No	Small	High	Unknown	Long	Reported
Schroeder 2020 [27]	Prospective; Internal	No	Pilot	Moderate	High-Risk Only	Standard	Reported
Støen 2017 [17]	Prospective; Internal	No	Limited	High	High-Risk Only	None	Reported
Verhage 2024 [54]	Prospective; Internal	No	Small	Moderate	High-Risk Only	None	Reported
Von Gunten 2023 [52]	Prospective; Internal	No	Pilot	Moderate	High-Risk Only	Short	Reported

## Data Availability

All studies included in this review are publicly available academic publications. Extracted and synthesized data are presented within the Supplementary Materials.

## Supplementary Materials

The following supporting information can be downloaded at: https://www.mdpi.com/article/10.3390/jcm15041510/s1, Table S1: Summary of All Included Studies.

## Abbreviations

The following abbreviations are used in this manuscript:

CP	Cerebral Palsy
GM	General Movements
GMA	General Movements Assessment
NDD	Neurodevelopmental Disorder
ASD	Autism Spectrum Disorder
FM	Fidgety Movements
SMA	Spinal Muscular Atrophy
