# Supplementary material for: Video and Wearable Sensor Technologies for Early Detection of Cerebral Palsy in Infants: A Scoping Review
Source: J Clin Med. 2026 Feb 14;15(4):1510. doi: 10.3390/jcm15041510 (PMC12941449; doi:10.3390/jcm15041510)
Supplement: Supplementary file 1 [file jcm-15-01510-s001.zip › jcm-4087522-supplementary/jcm-4087522-supplementary.pdf]

Table S1. Summary of Included Literature

Author (Year)	Country	Study design	Patient Population	Total N	Age (mean ± SD or range)	Pathology	Evaluation method	Follow-up time/duration	Primary Outcome(s)	Results	Data analysis
**Meinecke (2006)**	Germany	Prospective cohort	Full term, Preterm	22	N/A	CP	Video capture	2 years	Classification accuracy of motion analysis-based model for infantile CP risk	Overall detection rate of 73%	Computational algorithm
**Adde (2009)**	Norway	Prospective cohort	Full term, Preterm	82	10 to 18 weeks	CP	Video capture	N/A	Classification accuracy of GM Toolbox for FMs	Sensitivity: 81.5% Specificity: 70%	Computational algorithm
**Sgandurra (2009)**	Italy	Prospective cohort	Full term	7	4 to 9 months	Infantile neurological disorders	Video capture	N/A	Feasibility of measuring infants' grasping actions and manual forces in a natural setting using a sensorized biomechatronic gym with instrumented toys	The system quantified grasping forces and identified developmental trends in manual force over time	Computational algorithm
**Adde (2010)**	Norway	Prospective cohort	Preterm	30	10 to 15 weeks	CP	Video capture	5 years	Predictive value of computer-based video analysis for the development of CP	Sensitivity: 85% Specificity: 71%	Computational algorithm
**Adde (2013)**	Norway	Prospective cohort	Full term, Preterm	52	9 to 17 weeks	CP	Video capture	2 years	Detection of FMs; prediction of CP at 2 years	Absent FMs in children who developed CP; identified 100% of children who developed CP	Computational algorithm
**Rahmati (2014)**	Norway	Retrospective cohort	Not disclosed	78	N/A	CP	Video capture	2 to 5 years	Prediction of CP based on motion data	Sensitivity: 86% Specificity: 92% Accuracy: 91%	Computational algorithm
**Philippi (2014)**	Germany	Prospective cohort	Full term, Preterm	67	2.5 to 3.5 months (corrected)	CP	Video capture	2 years	Prediction of CP and neurodevelopmental impairment based on kinematic analysis of general movements	PPV: 0.75, CI 0.63–0.84	Computational algorithm
**Valle (2015)**	Norway	Prospective cohort	Full term	75	9 to 18 weeks	CP	Video capture	N/A	Test-retest reliability of a computer-based video analysis for quantitative assessment of general movements	Intraclass Correlation Coefficient (ICC) for Centroid of motion standard deviation: 0.80; Quantity of motion mean: 0.80;	Computational algorithm

										Quantity of motion standard deviation: 0.86	
**Støen (2017)**	Norway	Prospective cohort	Preterm	150	24 to 32.1 weeks	CP	Video capture	N/A	Detection of absent or sporadic FMs as a predictor of CP based on computer-based video analysis	Sensitivity: 90%<br>Specificity: 80%	Computational algorithm
**Khan (2018)**	Germany	Prospective cohort	Not disclosed	10	2 weeks to 6 months	Infantile movement disorders	Video capture	N/A	Accuracy and feasibility of a markerless, video-based method for detecting and tracking infants' body-part movements to support early identification of movement disorders	Markerless method outperformed existing techniques in infant body-part tracking on a large dataset	Machine learning
**Adde (2018)**	Norway	Prospective cohort	Preterm	27	3 to 15 weeks	CP	Video capture	N/A	Quantification of movement characteristics (FMs vs writhing general movements) using computer-based video analysis	Mean variability of the centroid of motion was 7.5% lower during fidgety GMs compared with writhing GMs (p = 0.004)	Computational algorithm
**Orlandi (2018)**	Canada	Retrospective cohort	Preterm	127	3 to 5 months	CP	Video capture	N/A	Prediction of CP using video-based analysis of infant movements.	Accuracy: 92%	Machine learning
**Ihlen (2019)**	Norway	Prospective cohort	Preterm	377	9 to 15 weeks	CP	Video capture	3.7 years	Accuracy of the Computer-based Infant Movement Assessment model in predicting a diagnosis of CP from infant video recordings	Sensitivity: 92.7%<br>Specificity: 81.6%	Computational algorithm
**Raghuram (2019)**	Canada	Retrospective cohort	Preterm	152	IQR 24.4 - 27.7 weeks	CP	Video capture	N/A	Prediction of motor impairment based on automated movement analysis from video recordings	Sensitivity: 79%<br>Specificity: 63%<br>Positive predictive value: 37%; Negative predictive value: 91%<br>Accuracy of GMA: 66%	Computational algorithm
**Schroeder (2020)**	Germany	Prospective cohort	Preterm	29	14.8 ± 0.7 weeks (corrected)	CP	Video capture	12 to 31 months	Agreement between automated 3D full-body based GMA and traditional expert-based GMA in predicting CP	GM-complexity: good-moderate (κ = 0.58; ICC = 0.874 [95%CI 0.730; 0.941])<br>Fidgety movements:	Computational algorithm

										substantial-good (κ = 0.78, ICC = 0.926 [95%CI 0.843; 0.965])	
**Passmore (2020)**	Australia	Retrospective cohort	Full term; Preterm	510	N/A	CP	Video capture	N/A	Agreement between machine learning-based 3D infant pose estimation from smartphone videos and expert GMA ratings.	90% of key-points were identified	Deep learning
**Caruso (2020)**	Italy	Retrospective cohort	Full term; Preterm	103	10 days to 36 months	NDD and ASD	Video capture	6, 12, 18, and 24 weeks	Differences in longitudinal motor trajectories, quantified by MOVIDEA-derived kinematic features, between infants at high risk for ASD and NDD and typically developing infants.	Early developmental trajectories of specific motor parameters were different in high-risk infants later diagnosed with NDD from those of infants developing typically	Computational algorithm
**Baccinelli (2020)**	Italy	Prospective cohort	Full term; Preterm	90	12 to 24 weeks	NDD	Video capture	6, 12, 18, 24, and 36 months	Reliability and operator-independence of motion feature extraction from single-camera infant videos using Movidea	Inter-operator reliability: ICC > 0.75<br>Trajectory tracking reliability: correlation coefficients 0.964–0.992<br>Mean information loss (2D vs 3D): 36.7%	Computational algorithm
**Tsuji (2020)**	Japan	Prospective cohort	Full term	21	2 to 20 weeks	NDD	Video capture	N/A	Accuracy of a markerless, video-based system for quantitative evaluation and classification of infant GMs compared with expert visual assessment by licensed clinicians	Mean agreement: 83.1% ± 1.84%<br>Classification accuracy for normal vs abnormal movements: 90.2% ± 0.94%.	Machine learning
**Blaschek (2021)**	Germany	Prospective cohort	Not disclosed	10	17 months	SMA	Video capture	N/A	Correlation between KineMAT-derived whole-body motion parameters and motor function (CHOP INTEND score) in children with SMA	4/5 predefined KineMAT motion parameters demonstrated very strong correlations with CHOP INTEND scores (r = 0.923–0.959) and low test–retest variability (1.3% to 10.5%)	Computational algorithm

**Reich (2021)**	Germany	Prospective cohort	Full term	45	35 to 41 weeks	NDD	Video capture	16 weeks	Accuracy of machine learning algorithm to detect age specific FMs	Classification accuracy: 88%	Machine learning
**Nguyen-Thai (2021)**	Australia	Retrospective cohort	Not disclosed	235	14 to 15 weeks	CP	Video capture	N/A	Performance of a pose-based, computer vision method for automated assessment of FMs in infants to support early CP screening from smartphone video using the BabyMoves App	ROC-AUC score: 81.87%	Machine learning
**Balta (2022)**	United States	Prospective cohort	Not disclosed	8	3 to 5 months	NDD	Video capture	5 months	Ability to derive quantitative GM metrics based on home-recorded, markerless 3D motion tracking (RGB-D sensor) to reflect clinically assessed GM quality in infants	8 established GM metrics plus one novel metric computed;<br>GM metrics demonstrated qualitative concordance with clinician evaluation	Deep learning
**Doi (2022)**	Japan	Prospective cohort	Not disclosed	41	4 months	ASD	Video capture	18 months	Analyze the relationship between spontaneous bodily movements at 4 months of age and the ASD risk at 18 months of age	(i) Reduced lower-limb movement strength and impaired upper–lower body balance, and<br>(ii) Higher central frequency with reduced spatial variability of medial–lateral body center motion,<br>were predictive of ASD risk at 18 months of age	Machine learning
**Groos (2022)**	Norway	Randomized controlled trial	Not disclosed	557	9 to 18 weeks	CP	Video capture	12 months	Assess the external validity of a novel deep learning-based method to predict CP based on videos of infants' spontaneous movements	Sensitivity: 71.4%<br>Specificity: 94.1%<br>Accuracy: 90.6%	Deep learning
**Raghuram (2022)**	Canada	Prospective cohort	Preterm	252	26 to 29 weeks	CP	Video capture	2 years	Validate automated movement analysis model that predicts CP in very preterm infants based on	Sensitivity: 55%<br>Specificity: 80%<br>Positive predictive value: 26%	Computational algorithm

									video recordings from fidgety movements	Negative predictive value: 93%	
**Moro (2022)**	Italy	Prospective cohort	Preterm	142	29.0 ± 2.0 weeks	CP	Video capture	30 months	Evaluate automated, markerless video analysis deep-learning model to detect motion patterns in spontaneous movements of preterm infants	Maximum accuracy: 85.7%	Deep learning
**Prosser (2022)**	United States	Prospective cohort	Full term; preterm	15	4 to 6.5 months	CP	Video capture	2 years	Compare biomechanical measures of early postural control between infants who go on to develop future impairment in motor control versus typically developing peers	Center of pressure path length significantly higher in infants who go on to impaired motor control ($p = 0.033$)	Computational algorithm
**Celik (2023)**	Turkey	Prospective cohort	Full term; preterm	101	9 to 20 weeks	NDD	Video Capture, Force Plate	N/A	Develop a quantitative assessment method of spontaneous movement using center-of-pressure (COP) movement analysis	Prediction of GMs: Sensitivity: 85% Specificity: 83% Accuracy: 83% Negative predictive value: 97%	Computational algorithm
**Abbasi (2023)**	Australia	Prospective cohort	Full term; preterm	15	24 to 41 weeks	NDD	Video capture	N/A	Validate automated motion analysis using markerless motion tracking technology on clinically recorded videos in infants	Overall tracking accuracy: 98.28% (SD 2.29) across 24 landmarks	Deep learning
**Passmore (2024)**	Australia	Prospective cohort	Full term; preterm	330	12 to 18 weeks (corrected)	NDD	Video capture	2 years	Quantify spontaneous infant movements using pre-trained deep learning model to track infant body parts on video	Increased high-velocity whole-body movement states were associated with abnormal/absent GMs ($p=0.003$), prematurity ($p \leq 0.001$), and lower 2-year motor outcomes ($p = 0.014$)	Deep learning
**Turner (2024)**	UK	Prospective cohort	Not disclosed	12	3 to 12 months	NDD	Video capture	N/A	Evaluate a transformer-based deep-learning fusion model that integrates multiple video features within a unified deep	Accuracy: 84% (single modality); 90.1% (multimodal)	Deep learning

									neural network to classify and analyze infant movement patterns		
**Passmore (2024)**	Australia	Prospective cohort	Full term; preterm	341	N/A	CP	Video capture	2 years	Validate pre-trained deep learning model to track infant body parts on video smart phone video taken at home by parents and predict GMs classification	Body point labelling approach achieved human-level accuracy (mean ± SD error of 3.7 ± 5.2% of infant length) Sensitivity (predicting expert GMs): 76 ± 15% Negative predictive value of 94% ± 3%	Deep learning and machine learning
**Berger (2019)**	United States	Prospective cohort	Full term; preterm	31	6-8.5 months	CP	Video capture	N/A	Ability of a novel video assessment to quantify trunk and pelvic movement during early sitting and to differentiate infants with typical development (TD), preterm motor delay, and CP under focused (FA) versus non-focused attention (NFA) conditions	Infants with TD and CP made fewer trunk movements during periods of FA than NFA ($p < 0.01$); Preterm infants exhibited more trunk/pelvic movement than other groups and did not differ based on attention type	Computational algorithm
**Ohgi (2008)**	Japan	Prospective cohort	Preterm	14	1 month	Brain Injury	Wearable accelerometer	N/A	Ability of triaxial accelerometer to measure characteristics of spontaneous upper-extremity movements of premature infants with brain injuries and those without brain injuries	Movements of infants with brain injury had significantly higher movement randomness (Lyapunov exponent) and complexity (Optimal Embedding Dimension) than controls ($p < 0.05$ for both)	Computational algorithm
**Abrishami (2019)**	United States	Prospective cohort	Full term; preterm	36	2 to 15 months	CP	Wearable accelerometer	24 months	Ability of wearable sensors to differentiate between infants with typical development and those with or at risk for developmental delays	Full day sampling (as opposed to shorter sessions) showed significant group differences in movements across typically developing and at-risk infants:	Computational algorithm

										Duration: p = 0.008<br>Peak acceleration: p = 0.03<br>Mean acceleration: p = 0.04	
**Fontana (2021)**	Italy	Prospective cohort	Full term; preterm	68	N/A	NDD	Wearable accelerometer	N/A	Quantify abnormal movements of the limbs at term equivalent age (TEA) using an accelerometer-based method	Identifying infants with abnormal movements:<br>Sensitivity: 88%<br>Specificity: 86%	Computational algorithm
**Franchi De Cavalieri (2023)**	Italy	Prospective cohort	Full term; preterm	17	6.35 ± 2.95 months	CP	Tri-Axial Accelerometer	8 weeks	Predict clinical assessment scores of infants' motor activity in high-risk infants based on accelerometry data	Achieved high explanatory power: Adjusted R^2: 0.98 (Infant Motor Profile total); 0.99 (Alberta Infant Motor Scale)	Computational algorithm
**Von Gunten (2023)**	Switzerland	Prospective cohort	Full term; preterm	8	9 to 12 months	CP	Wearable accelerometer	6 weeks	Feasibility of Action Observation Training (AOT) in combination with sensor-based measurements in infants at high risk of Unilateral Spastic CP (UCP)	AOT was feasible and associated with significant short-term improvements in hand function. Sensor measures correlated strongly with clinical assessments	Computational algorithm
**Bard-Pondarré (2023)**	France	Single case experimental design	Not disclosed	6	3 to 12 months	CP	Wearable accelerometer	3 to 4 months	Evaluate ability of accelerometry to quantitatively assess hand function in comparison to Hand Assessment for Infants (HAI) in infants at risk of unilateral CP	Wrist-worn accelerometers were well tolerated and feasible. However, no strong correlation between accelerometry parameters and HAI scores	Computational algorithm
**Airaksinen (2023)**	Finland	Prospective cohort	Not disclosed	116	4.1 to 19.5 months	Typical Neurodevelopment	Wearable movement monitor	10 months	Validate performance of a wearable system for quantitative assessment in early motor development and compare to developmental tracking of physical growth chart - generating	Motor DAP scores explained:<br>97–99% of variance at the group level<br>80–82% of variance at the individual level	Deep learning

									Developmental Age Prediction (DAP) score.		
**Verhage (2024)**	Netherlands	Prospective cohort	Full term; preterm	50	3 to 12 months	CP	Wearable accelerometer	N/A for outcome of interest	Evaluate the ability of accelerometry-derived measures to quantitatively assess asymmetry in upper limb activity in infants aged 3–12 months who are at risk for developing unilateral spastic cerebral palsy (UCSP)	High discrimination (AUC: 0.88–0.96); Strong Hand Assessment for Infants correlation (r: 0.61–0.84).	Computational algorithm
**Karch (2008)**	Germany	Prospective cohort	Full term; preterm	20	0 to 3 months	NDD	Wearable sensors	N/A	Validation of method to capture the movements of infant extremities using an electromagnetic tracking system and to reliably calculate the segmental kinematics	Abnormal GM reflected in reduced variability and stereotyped kinematics	Computational algorithm
**Karch (2012)**	Germany	Prospective cohort	Not disclosed	75	3 months	CP	Wearable Sensors	2 years	Quantitative assessment of stereotyped movements in order to inform prognosis of neurologic deficits	Sensitivity: 90% Specificity: 96%	Computational algorithm
**Airaksinen (2020)**	Finland	Prospective cohort	Not disclosed	22	4.5 to 7.7 months	NDD	Wearable sensors	N/A	Validation of an infant wearable, a multi-sensor smart jumpsuit that allows mobile accelerometer and gyroscope data collection during movements	Performance (CNN classifier): Posture classification accuracy: 99.1% Movement classification accuracy: 90.7% (full-agreement frames)	Deep learning
**Wilson (2021)**	USA	Prospective Cohort	Not disclosed	5	3,6,8, and 12 months	ASD	Wearable sensors	36 months	Development of a measure of motion complexity based on data from Opal wearable sensors and evaluation of its relationship to developmental outcomes in high-risk infants	HR infants later diagnosed with ASD had lower motion complexity compared to HR infants who did not develop ASD; Motion complexity correlated more strongly with later ASD diagnosis than with measures of	Computational algorithm

										cognitive ability or adaptive skills	
**Barrois (2024)**	France	Prospective cohort	Not disclosed	25	3 to 20.9 months	SMA	Wearable sensors	2 years	To evaluate the acceptability, validity and clinical relevance of IMU to monitor spontaneous movement recovery in early onset SMA patients after GT	IMU-derived movement amplitude metrics: ICC 0.85–0.93<br>Correlation with CHOP INTEND ($r = 0.61–0.74$, $p < 0.001$) Increase in metrics over 12 months ($p \leq 0.006$)	Computational algorithm
**Rahmati (2016)**	Norway	Prospective cohort	Not disclosed	78	10 to 18 weeks	CP	IMU	Up to 5 years	Validate frequency analysis and feature reduction method for early CP prediction	Sensitivity: 85%<br>Specificity: 92%<br>Accuracy: 91%	Machine learning
**Kuo (2022)**	United States	Prospective cohort	Full term	12	3 to 9 months	Developmental Delay	Hand use and grasp sensor	6 months	Infant grasp frequency and duration, peak grasping force, average grasping force, force coefficient of variation, and proportion of bimanual grasps	Average grasp force, accumulated grasp time, Bimanual grasps all increased significantly with age (all $P < 001$).	Computational algorithm

Abbreviations: Cerebral Palsy (CP); General Movements (GM); General Movements Assessment (GMA); NDD (NDD) Autism Spectrum Disorder (ASD); Fidgety Movements (FM); Spinal Muscular Atrophy (SMA); Inertial Measurement Units (IMU); Interclass Correlation Coefficients (ICC)
